# Performance of Five Postharvest Storage Methods for Maize Preservation in Northern Benin

**DOI:** 10.3390/insects11080541

**Published:** 2020-08-17

**Authors:** Dieudonne Baributsa, Ousmane Nouhou Bakoye, Baoua Ibrahim, Larry L. Murdock

**Affiliations:** 1 Department of Entomology, Purdue University, West Lafayette, IN 47907, USA; murdockl@purdue.edu; 2 Department of Science and Techniques of Plant Production, Dan Dicko Dankoulodo University of Maradi, Maradi BP 465, Niger; ousmanebakoy@yahoo.fr (O.N.B.); baoua.ibrahim@gmail.com (B.I.)

**Keywords:** insect pests, grain losses, hermetic storage bags, Sahel, West Africa

## Abstract

**Simple Summary:**

Smallholder farmers in sub-Saharan Africa lose grain to insect pests during storage. To reduce these losses, several storage technologies are available for sale in East Africa; but only a few are marketed in West Africa. We assessed the performance of four types of hermetic bags (SuperGrainbagTM, AgroZ^®^ bag, EVALTM, and Purdue Improved Crop Storage-PICSTM bags), as well as an insecticide-treated woven bag (ZeroFly^®^), and a polypropylene (PP) woven bag. The trials sought to determine if these technology packages prevent losses of insect-infested maize purchased in local markets in northern Benin. After seven months of storage, we found that maize stored in hermetic bags did not suffer further damage or lose weight due to insects. By contrast, grain stored in insecticide-treated and regular PP woven bags had weight losses of 6.3% and 10.3%, respectively. Grain moisture content of maize kept in hermetic bags remained unchanged during the 7-month storage period. However, moisture content decreased by about 30% in insecticide-treated and PP woven bags due to the prevailing dry environmental conditions. Farmers and development agencies in the Sahel can use and/or recommend these hermetic technologies to reduce maize grain storage losses due to insects.

**Abstract:**

Several postharvest technologies are currently being commercialized to help smallholder farmers in sub-Saharan Africa reduce grain storage losses. We carried out a study in Northern Benin to compare the effectiveness of five technologies being sold to protect stored grain. Maize that had been naturally infested by insects was stored in four hermetic storage technologies (SuperGrainbag™, AgroZ^®^ bag, EVAL™, and Purdue Improved Crop Storage-PICS™ bags), an insecticide impregnated bag (ZeroFly^®^), and a regular polypropylene (PP) woven bag as control. Oxygen levels in hermetic bags fluctuated between 0.5 ± 0.0 (*v*/*v*) and 1.0 ± 0.3 (*v*/*v*) percent during the seven months of storage. No weight loss or insect damage was observed in grain stored in any of the hermetic storage bags after seven months. However, grain stored in ZeroFly^®^ and PP woven bags had weight losses of 6.3% and 10.3%, respectively. These results will help farmers and development agencies when making decisions to use and/or promote storage technologies to reduce postharvest grain losses.

## 1. Introduction

Maize is a major staple food crop in several countries in sub-Saharan Africa. Significant crop losses can occur during postharvest handling and storage. Maize postharvest losses vary significantly by crop, stage in the value chain, and geography but may reach up to 20% or higher [1,2,3,4,5,6]. Losses are primarily caused by feeding associated with population growth of pests (particularly insects, rodents, etc.) and the presence of mycotoxins [7,8,9,10]. To deal with postharvest losses during storage, farmers employ a variety of measures including traditional methods, applying chemicals, or selling their grain soon after harvest. Many of the storage methods used by farmers have challenges. These include limited access and cost-effectiveness, lack of scalability, and in some cases, they are not adapted to local situations. Selling grain right after harvest results in loss of potential income and food insecurity at the household level. Some smallholder farmers sell most of their grain due to households’ needs for cash after harvest [11], resulting in food insecurity in subsequent months. Often, grain prices significantly increase (e.g., may double) from harvest to lean season [12,13]. Hence, storing grain provides an opportunity to be food secure but also allows farmers to tap into better grain prices.

Hermetic storage is an ancient technology that has received renewed attention in recent decades and has led to development of modern hermetic storage technologies (HST). These HSTs have been used to preserve grain and seed during storage [14,15,16]. Hermetic technologies encompass rigid containers such as plastic and metal silos and drums as well as collapsible containers such as hermetic bags. In the last two decades, hermetic grain storage bags have been tested and promoted around the world, but their scale-up began in West and Central Africa with the widespread dissemination of the Purdue Improved Crop Storage (PICS™) [12,17]. The PICS™ bags were first promoted for cowpea storage [14] but later on were proven to be effective in storing a variety of crops including maize, common beans, rice, sorghum, Bambara nuts, and mung beans [7,8,18,19,20]. 

The strategy used to scale-up the PICS™ technology in West and Central Africa attracted private sectors, governments, and donors to invest in the development and/or the dissemination of hermetic technologies. The SuperGrainbag™, a single layer hermetic bag promoted in Asia for rice storage, was introduced into the African market in the late 2000s by GrainPro [21]. In recent years, several other hermetic storage bags were introduced to smallholder farmers in Africa, including AgroZ^®^ (manufactured by A to Z in Arusha, Tanzania) and Elite (manufactured by Elite Innovations in Eldoret, Kenya) bags [13,22,23]. In addition, Kuraray Co. Ltd. (Osaka, Japan) began exploring markets for its EVAL™ bags in the African market. Several studies have been conducted to assess the efficacy of different hermetic bags for storage of grains in different countries and regions of the world [18,24,25,26,27,28,29]. Limited studies have looked at comparing the different hermetic bags available in sub-Saharan Africa. A side-by-side experiment comparing PICS™ and SuperGrainbag™ for storing cowpea in Niger found no difference in effectiveness between the two hermetic bags [30]. Additional studies comparing several hermetic bags for controlling *Prostephanus truncatus* (Horn, Coleoptera: Bostrichidae) on stored maize in Malawi, Tanzania, and Zimbabwe found no difference among these technologies [22,28,31]. These hermetic bags are as effective as and often better than insecticides in protecting grains against insect attacks during storage [24,27,32]. 

More brands of hermetic bags are becoming commercially available due to the growing demand among smallholder farmers and the interest of the private sector in the business. In Kenya, more than five brands of hermetic bags are being commercialized, including PICS™, AgroZ^®^, SuperGrainbag™, ZeroFly^®^, and Elite bags [13]. Increased availability of various brands of hermetic bags is beneficial to farmers and other users of these technologies. Before these hermetic bags are fully commercialized in the Sahel region, there is a need to assess their performance in field conditions. Do they all work, and equally well? If so, then other considerations need attention, including cost, availability, and durability. We undertook this study to compare side-by-side performance of bag storage technologies available in East Africa for preserving maize in the Sahel. The findings of the study should be useful for farmers, grain traders, development agencies, and governments in decision-making for postharvest maize preservation. 

## 2. Materials and Methods

The experiment was conducted in Parakou, Benin in collaboration with a group of maize traders. The trials were initiated on 14 August 2016 during the rainy season and were ended on 19 March 2017 during the dry season (218 days or about seven months). Five types of storage technologies were tested, including PICS™, SuperGrainbag™, AgroZ^®^, EVAL™, and ZeroFly^®^ bags. Conventional polypropylene (PP) woven bags were used as controls. The characteristics of the various bags used in this experiment are described in Table 1. They include brand, composition, thickness, and supplier of bags (where the bags were purchased or donated from). The capacity of the bags used in this experiment ranged from 50 to 100 kg. The bags were filled with 50 kg of naturally-infested maize purchased locally. All the maize was thoroughly mixed to ensure homogeneity before filling the bags. All bags were closed using strings as described in an extension training guide [33]. Each treatment was replicated four times. The bags were kept in a trader’s warehouse for the duration of the trial.

The infestation level in the maize was assessed at the beginning of the experiment and after seven months. Twelve 500 g samples (*n* = 12) were randomly collected from each treatment (three samples per replicate). Each 500 g sample was sieved to separate and count live adults of each insect species. Three sub-samples of 100 grains were randomly taken from each of the three 500 g samples, resulting in 900 grains per replicate (*n* = 36 per treatment). These 100 grain sub-samples were used to assess insect damage and weight loss. We calculated the insect-damaged grain [34] and the weight losses [35] using the formulas below:

$$\%\;\mathrm{Insect}-\mathrm{damaged}\;\mathrm{grain}=\frac{\left(\mathrm{Number}\;\mathrm{of}\;\mathrm{damaged}\;\mathrm{grains}\right)}{\mathrm{Total}\;\mathrm{grain}\;\mathrm{count}}\times \;100$$

$$\%\;\mathrm{weight}\;\mathrm{loss}=\frac{\left(\mathrm{DWo}-\;\mathrm{DWt}\right)}{\mathrm{DWo}}\times \;100$$

where DWo is dry weight at the beginning, and DWt is dry weight at the end. The DW was obtained using the following formula $\mathrm{DW}=\mathrm{Weight}\frac{\left(100-\mathrm{MC}\right)}{100}$ where MC is the moisture content of the sample.

Grain moisture content was measured using the Dickey John mini GAC (DICKEY-john, Auburn, IL, USA). Three measurements were made for each replicate (12 measurements for each treatment). Oxygen and CO_2_ concentrations in each bag were recorded one day after closing the bags and just before opening them using a Mocon PAC Check Model 325 Headspace analyzer (Mocon, Minneapolis, MN, USA) fitted with a 20-gauge hypodermic needle for sampling through the walls of the storage bags. The puncture hole in the outer bag was sealed with plastic tape after each measurement. Data loggers, EL-USB-2 model (Lascar, Whiteparish, Wiltshire, UK), were placed in one bag for each treatment to record temperature and relative humidity during the duration of the experiment. The data logger recorded temperature and relative humidity every one hour. Data presented in the graphs are daily averages. Upon completion of the experiment, the liners of hermetic bags were inspected for perforations (holes) made by insects.

Microsoft Excel was used to calculate means and standard errors of means. For both temperatures and relative humidity, daily averages were calculated. The statistical analysis was done with SPSS 24.0 (IBM Corporation, Armonk, NY, USA). The analysis of variance (ANOVA) followed by least significant difference (LSD) was used to compare means of oxygen and carbon dioxide concentrations, weight loss, infestation levels, and damage per treatments.

## 3. Results

The maize used in this experiment had an initial mean infestation of 52.5 insects per 500 g of which 50.0% were *Sitophilus zeamais* Motschulsky (Coleoptera: Dryophthoridae), 20.5% were *P. truncatus*, and 29.5% were other insects (*Rhyzopertha dominica* (Fabricius) (Coleoptera: Bostrichidae), *Tribolium castaneum* (Herbst) (Coleoptera: Tenebrionidae), and *Cryptolestes ferrugineus* Stephens (Coleoptera: Laemophloeidae)) (Table 2). After seven months of storage, no live insects were observed in any of the hermetic bag treatments. However, in the PP woven and the ZeroFly^®^ storage bags, insect populations ranged from 1.8 to 5.3 insects per 500 g for S. *zeamais, R. dominica, T. castaneum*, and *C. ferrugineus*. No *P. truncatus* population was observed in PP woven bags and ZeroFly^®^ storage bag treatment at the end of the experiment. 

After seven months of storage, the average grain moisture content (MC) in grain stored in hermetic containers did not differ from that observed at the beginning of the experiment. By contrast, grain stored in the PP woven bag and in the ZeroFly^®^ storage bag exhibited decreased average moisture content by 4.3% (Table 3).

**Figure 1:**
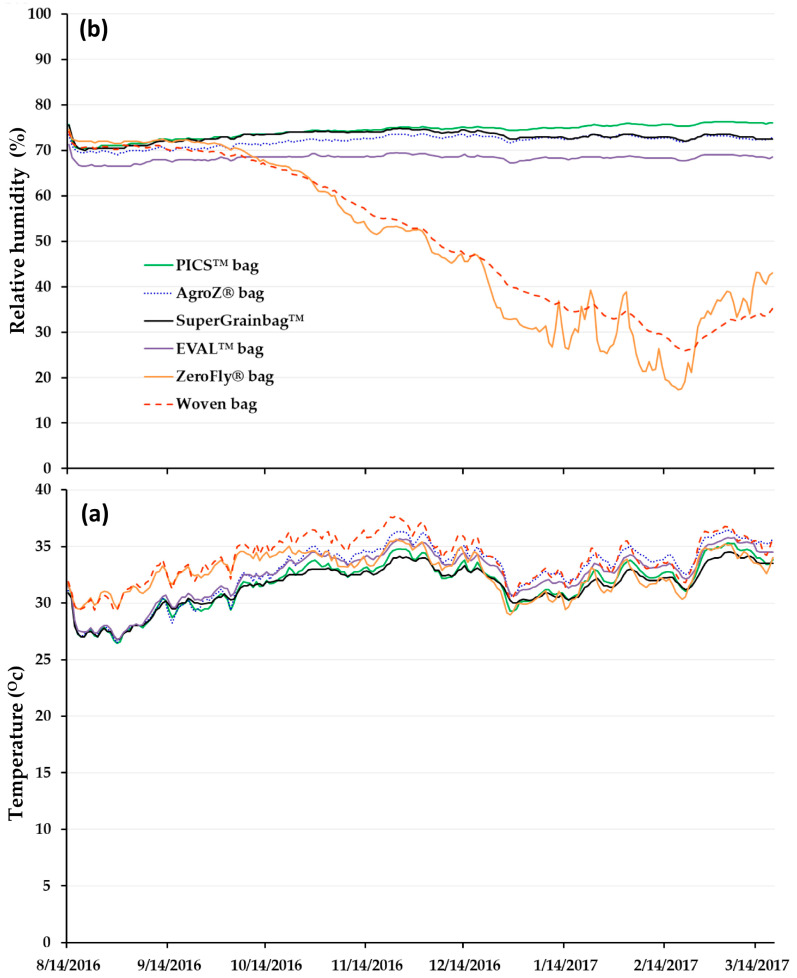
(**a**) Daily average temperature and (**b**) relative humidity variation in six types of storage bags filled with naturally infested maize stored for seven months in Parakou, Benin.

A week after the launch of the experiment (21 August 2016), the average daily temperature was higher in the woven PP bags and the ZeroFly^®^ bags compared to the other treatments (Figure 1a). Temperatures in the woven PP and in the ZeroFly^®^ bags varied between 30.3 ± 0.0 and 30.6 ± 0.0 °C, respectively, and between 27.5 ± 0.0 and 27.8 ± 0.0 °C for the hermetic bags (*F* = 1093.0, 5/138, *p* < 0.001). Temperatures in the PP woven bags reached their highest point around the third week of November 2017 at 37.7 ± 0.0 °C. Average temperatures were the highest in the ZeroFly^®^ bags and the four hermetic storage methods around the same period. Towards the end of the experiment (5 March 2017), the mean daily temperatures were different among the six treatments ranging from 34.5 ± 0.0 to 36.8 ± 0.1 °C (*F* = 77.57, 5/138, *p* < 0.001).

Three days after setting up the experiment, daily average relative humidity differed among the six storage containers ranging from 72.0 ± 0.0 to 66.9 ± 0.0% (*F* = 2267.00, 5/138, *p* < 0.001) (Figure 1b). Toward the third week of October, about two months after the experiment was set up, the relative humidity in both the PP woven and the ZeroFly^®^ bags was already trending lower. On the other hand, the relative humidity in all hermetic bags remained relatively constant. Around 5 March 2017 (toward the end of the experiment), relative humidity remained lower in the woven bag and the ZeroFly^®^ bags ranging between 32.2 ± 0.2 and 39.0 ± 0.2%, but there was an upward trend.

One day after the launch of the experiment, oxygen levels in the hermetically sealed bags drastically decreased from 21% to levels between 0.8% to 2.3% but were not significantly different among the different hermetic bags (Table 4). In the non-hermetic bags (ZeroFly^®^ and PP woven bags), oxygen levels remained at around 20% one day after closing the bags and stayed at this level until the end of the experiment. Oxygen levels in the ZeroFly^®^ bag treatment did not differ from that noted in the PP woven bag. The CO_2_ levels in each of the types of hermetic bags were not significantly different one day after the closure of the bags (12.6% to 14.0%) and seven months later (14.1% to 16.6%). The ZeroFly^®^ bag treatment recorded CO_2_ levels (below 0.5%) comparable to those observed in the PP woven bags during the two periods of observation.

After seven months of storage, we observed weight loss of 10.3% in PP woven and 6.3% in ZeroFly^®^ storage bag treatments, while there was no significant weight loss in any of the hermetic bags (Table 5). In addition, no grain damage beyond that which was originally present was observed in hermetically sealed bags while it more than doubled to 62.5% in the control PP woven bag and reached 53.3% in the ZeroFly^®^ storage bag treatments. At the end of the experiment, no perforations were observed on any inner liners of the hermetic technologies (Table 5). However, some alterations/abrasions (small holes) were observed on the liners of the hermetic bags; no significant difference was observed among treatments. No perforations or abrasions were observed on the middle (second) liner of the PICS™ bag.

## 4. Discussion

This study carried out in a trader’s warehouse in Benin established that maize has several post-harvest storage insect pests. The five insect pests that were present in maize grain at the trial set-up were *P. truncatus*, *S. zeamais*, *R. dominica*, *T. castaneum*, and *C. ferrugineus*. Three insects were predominant at the beginning of the experiment- *S. zeamais*, *P. truncatus*, *and C. ferrugineus*. However, after seven months of storage, all insects had died in hermetic bags while there were still live insects in the PP woven and the ZeroFly^®^ bags. Hermetic bags are known to be effective at suppressing insect development and reproduction in stored cereal and legume crops [7,8,18,24,36,37]. Our results corroborate findings of previous studies comparing several hermetic technologies in the Sahel and other regions in sub-Saharan Africa: SuperGrainbag™ and PICS™ bags in Niger for preserving cowpea [30]; PICS™, SuperGrainbag™, and AgroZ^®^ bags in Tanzania for storing maize [22]; PICS™ and SuperGrainbag™ for storing maize in Kenya [38]; PICS™ and SuperGrainbag™ for storing maize in Malawi [28]; and PICS™, SuperGrainbag™ and EVAL™ (Kuraray) bags in Zimbabwe for maize storage [29,31].

In the PP woven and the ZeroFly^®^ bags, *P. truncatus* died after seven months of storage; the other four insect pests were still found alive. The supply of oxygen in both PP woven bags and ZeroFly^®^ bags allowed the insect pests to continue to develop and reproduce and damage the grain. The present results are consistent with those of studies comparing hermetic and non-hermetic storage methods in Ghana, Malawi, Tanzania, and Zimbabwe, which demonstrated that ZeroFly^®^ did not control insect populations during the experiments [28,29,39,40]. The higher temperatures and the relative humidities observed during the first three months of the experiment favored insect growth, development, and reproduction and contributed to the losses observed in the non-hermetic technologies. Because they are porous to air, the grain held in PP woven and ZeroFly^®^ bags was affected by change in ambient relative humidity during storage. Low insect populations after seven months of maize storage could be explained by the significant decrease in relative humidity in both PP woven and ZeroFly^®^ bags treatments. The relative humidity was high during the rainy season and then dropped in the dry season. This low humidity may explain the low survival of *P. truncatus* seen with non-hermetic technologies (PP woven bag and ZeroFly^®^ bag), because this species develops best at high relative humidity [41]. The increase and the drop in relative humidity may also explain the decrease in moisture content of the maize grain in PP woven and ZeroFly^®^ bags after seven months of storage. As noted in previous studies, relative humidity and grain moisture content in the hermetic technologies are least affected by prevailing ambient conditions [42,43,44,45].

There was no grain damage and negligible weight loss of the maize grain stored in hermetic bags. In the control PP woven bag, there was an increase of 32.0% of grains with holes and 10.3% weight loss after seven months of storage. This weight loss, though substantial, was lower than the 20–50% reported in other studies after 6–6.5 months of storage of maize in Benin [7,46,47]. The grain damage and the weight loss in PP bags were due to the high level of insect infestation. Insects developed in the ZeroFly^®^ storage bag; however, the proportions of damaged grain and weight losses were 9.3 and 4.0 percentage points lower, respectively, compared to the control PP woven bag. This could be explained by the fact that ZeroFly^®^ bag yarns are impregnated with deltamethrin. This pesticide affects insects when they come into contact with the wall of the bag during storage. Our results corroborated findings of other studies that showed ZeroFly^®^ bags do not effectively protect grain from insect damage during storage [28,29,40]. Weight loss of 6.3% in this study was higher than 3.7% observed in Ghana [39], lower than the 7.2% in Malawi [28], but significantly lower than the 23% and the 35% observed in Zimbabwe [29] and Tanzania [40], respectively. The lower weight loss observed in grain stored in Zerofly bags in this study was presumably due to the reduction in insect population caused by lower relative humidity during storage.

The ZeroFly^®^ technology designed to prevent both internal and external insect infestation did not provide full grain protection but appeared to perform better than PP woven bags. Since these bags are being sold in several countries in sub-Saharan Africa for on-farm grain storage, it was important to assess their performance under field conditions. These results with ZeroFly^®^ bags make it necessary for farmers to disinfest their grain before storage, which is difficult to do under smallholder farmers’ conditions [28,29]. Access to and cost of fumigants is a challenge for many smallholder farmers. The knowledge and the use of simple technologies such as solar disinfestation [48] are still limited in many developing countries. Based on in-field efficacy studies conducted in several countries in Africa that have shown insects survival and damage of grain stored in ZeroFly^®^ bags [28,29,40], and despite a laboratory trial in Ghana suggesting otherwise [39], a new ZeroFly^®^ hermetic bag was developed by Vestergaard Frandsen Ltd. The ZeroFly^®^ hermetic bag is the ZeroFly^®^ bag fitted with a multilayered plastic liner inside to stop the development of insects inside the bags while the impregnated woven bag (with the pesticide deltamethrin) stops infestations from outside [49]. ZeroFly^®^ hermetic bags are now commercially available in Kenya and several other countries in sub-Saharan Africa [13]. Cases of insects making holes in hermetic bags from outside have been reported during an on-station experiment in Kenya and Malawi [27,28]; however, fewer farmers appear to report this as a major issue during storage [13]. This is due to the training provided to farmers on proper handling of hermetic bags during storage. Farmers are advised not to store hermetic bags together with PP woven or jute bags that contain highly infested grain to minimize insect damage on the liners of hermetic bags from outside [27,33]. 

We observed a substantial reduction in the oxygen content and an increase in CO_2_ in the hermetic bags compared to the non-hermetic ones. Regardless of the different design (single, double, or triple bags) and composition (monolayer or multilayer liner) of hermetic bags, all technologies exhibited the same oxygen levels. Hermetic conditions allowed the preservation of the initial moisture content level of the maize and the internal relative humidity during the seven months of storage. Maize grain can safely be stored in hermetic bags at a moisture content of 14% or below, as no mold growth or aflatoxin development were observed [50]. Because most smallholder farmers store grain that is often used as seed for planting, it is recommended that maize be stored in hermetic bags at 13% moisture content or below to preserve seed viability [26]. Hermetic conditions led to almost total mortality of the insect pests, thus preventing losses during storage; this has already been noted by several studies [7,8,22,24,29]. The lower temperatures observed in hermetic treatments during this experiment confirm the inactivity of insect pests and the effectiveness of these methods in preserving the maize grain.

Minor insect damage on liners of all hermetic bags were observed at the end of the storage period, but it was not significantly different across all the hermetic technologies. These deteriorations or blemishes were limited to scratching or possibly salivation due to insect pest activities. Similar findings have been highlighted by other studies in sub-Saharan Africa [22,28,30]. Insect damages on liners of single or double bags such as SuperGrainbag™, AgroZ^®^ and EVAL™ bags could affect their reuse. The second liner of the PICS™ bags provides extra protection in case of insect damage, given that most abrasions are limited to the inner most liner. To minimize damage on liners of hermetic bags, farmers are advised to store clean grain (right after harvest and drying) when the insect infestation is very low [33]. Other minor damages to liners such as small holes can be repaired with adhesive tape to increase the longevity of these hermetic bags. While studies have shown that triple layers (e.g., PICS™ bags) can be used to store grain during three seasons [13,51], no information is available on single and double bags.

Since the launch of the scale-up of the PICS technology in the Sahel 12 years ago, it remains the most commercially available hermetic bag to farmers in several countries [52]. Several projects and initiatives have promoted several brands of hermetic bags in Kenya to reduce storage losses through prize competition [23,53]. These efforts have provided the private sector with incentives to improve the availability of hermetic bags among smallholder farmers. In addition, competition has led to decreases in prices of hermetic bags (e.g., in 2014, a triple layer PICS™ bag was $3.00 USD while, in 2019, the price of same PICS™ bags and other hermetic bags was about $2.50 USD). Similar efforts to promote hermetic storage bags in West and Central Africa would help improve availability at the farm-level, provide farmers with more alternatives, and eventually lead to a reduction in prices of these technologies.

## 5. Conclusions

The results of this study show that hermetic bags SuperGrainbag™, AgroZ^®^ bag, EVAL™ bag, and PICS™ bag are effective for safe maize storage in the Sahel. ZeroFly^®^ hermetic bags (not tested in this study) instead of the non-hermetic ZeroFly^®^ storage bag would be preferred because most smallholder farmers do not disinfest their grains before storage. Increased awareness of all these hermetic bags among smallholder farmers and development partners (e.g., government, non-profit organizations (NGOs), projects) in West Africa would increase competition and eventually improve availability and reduce prices.

## Figures and Tables

**Table 1:** Characteristics of six storage bags tested during this experiment in Parakou Benin.

Hermetic Bags	Woven Bag (PP)	Liner *	Liner Thickness (µm)	Supplier
PICS™ bag	1 PP bag	2 high density polyethylene liners	80	Lela Agro Industries Ltd. Kano, Nigeria
AgroZ^®^ bag	1 PP bag	1 multilayer polyethylene liner	90	A to Z Textile Mills Ltd. Nairobi, Kenya
SuperGrainbag™	1 PP bag	1 multilayer polyethylene liner	78	GrainPro Inc., Ltd. Nairobi, Kenya
EVAL™ bag **	No PP bag	1 multilayer polyethylene liner	100	Kuraray Private Ltd., New Delhi, India
ZeroFly^®^ storage bag ***	1 PP bag	No liner	NA	Vestergaard Frandsen Ltd., Nairobi, Kenya
PP bags	1 PP bag	No liner	NA	Lela Agro Industries Ltd. Kano, Nigeria

* Bags with at least one liner are hermetic. ** In this experiment, EVAL™ liner was fitted with a PP woven bag to provide support and ease handling. *** Polypropylene bag is impregnated with insecticide Deltamethrin.

**Table 2:** Live insect population (number per 500 g) in naturally infested maize grain stored for seven months using six storage bags in Parakou, Benin.

Treatments	*n*	*P. truncatus*	*S. zeamais*	*R. dominica*	*T. castaneum*	*C. ferrugineus*
Initial infestation	72	10.8 ± 2.4 a	26.2 ± 4.0 a	3.8 ± 0.4 a	3.9 ± 0.9 a	7.9 ± 1.2 a
After 7 months						
PICS™ bag	12	0.0 ± 0.0 b	0.0 ± 0.0 b	0.0 ± 0.0 c	0.0 ± 0.0 b	0.0 ± 0.0 c
AgroZ^®^ bag	12	0.0 ± 0.0 b	0.0 ± 0.0 b	0.0 ± 0.0 c	0.0 ± 0.0 b	0.0 ± 0.0 c
SuperGrainbag™	12	0.0 ± 0.0 b	0.0 ± 0.00 b	0.0 ± 0.0 c	0.0 ± 0.0 b	0.0 ± 0.0 c
EVAL™ bag	12	0.0 ± 0.0 b	0.0 ± 0.0 b	0.0 ± 0.0 c	0.0 ± 0.0 b	0.0 ± 0.0 c
ZeroFly^®^ bag	12	0.0 ± 0.0 b	2.8 ± 0.8 c	1.7 ± 0.5 b	5.3 ± 0.8 a	2.3 ± 0.7 b
Woven bag	12	0.0 ± 0.0 b	3.2 ± 0.9 c	1.8 ± 0.5 b	5.2 ± 0.9 a	2.6 ± 0.7 b
ANOVA		*F* = 9.90; *df* = 6/89; *p* < 0.01	*F* = 19.23; *f* = 6/89; *p* < 0.01	*F* = 19.95; *df* = 6/89; *p* < 0.01	*F* = 12.80; *df* = 6/89; *p* < 0.01	*F* = 16.35; *df* = 6/89; *p* < 0.01

Means within the same column followed by the same letter are not significantly different (least significant difference (LSD) 5%).

**Table 3:** Moisture level of naturally infested maize stored in six types of storage bags for seven months in Parakou, Benin.

Treatments	*n*	Moisture Content (%; Mean ± Standard Error of the Mean)
Initial	72	13.4 ± 0.1 a
After 7 months		
PICS™ bag	12	13.4 ± 0.2 a
AgroZ^®^ bag	12	13.2 ± 0.1 a
SuperGrainbag™	12	12.8 ± 0.1 a
EVAL™ bag	12	13.2 ± 0.1 a
ZeroFly^®^ bag	12	9.1 ± 0.1 b
Woven bag	12	9.1 ± 0.1 b
ANOVA		*F* = (150.11; *df* = 6/137; *p* < 0.01)

Means in the same column followed by the same letter are not significantly different (LSD 5%).

**Table 4:** Levels of O_2_ and CO_2_ in six types of storage bags filled with naturally infested maize stored for seven months in Parakou, Benin.

		Level of GAS in Bags (%; Mean ± Standard Error of the Mean)			
		O_2_		CO_2_	
Storage Method	*n*	1 Day After Bag Closure	7 Months After Bag Closure	1 Day After Bag Closure	7 Months After Bag Closure
PICS™	4	0.8 ± 0.3 a	0.7 ± 0.4 a	13.3 ± 0.2 a	16.2 ± 0.6 c
AgroZ^®^ bag	4	2.3 ± 0.9 a	0.7 ± 0.1 a	13.7 ± 0.3 a	16.1 ± 0.6 c
SuperGrainbag™	4	1.0 ± 0.3 a	0.5 ± 0.1 a	12.6 ± 0.9 a	14.1 ± 0.1 a
EVAL™ bag	4	0.8 ± 0.2 a	0.5 ± 0.0 a	14.0 ± 0.12 a	16.6 ± 0.4 c
ZeroFly^®^ bag	4	20.3 ± 0.1 b	20.04 ± 0.2 b	0.3 ± 0.1 b	0.5 ± 0.1 b
Woven bag	4	20.3 ± 0.1 b	20.3 ± 0.06 b	0.0 ± 0.0 b	0.3 ± 0.0 b
		*F* = 589.26; *df* = 5/18; *p* < 0.01	*F* = 4149.00; *df* = 5/18; *p* < 0.01	*F* = 295.25; *df* = 5/18; *p* < 0.01	*F* = 435.35; *df* = 5/18; *p* < 0.01

Means within the same column followed by the same letter are not significantly different (LSD 5%).

**Table 5:** Grain damage and weight of naturally infested maize as well as number of abrasions on liner of hermetic bags after seven months of storage in Parakou, Benin.

Treatments	*n*	100 Grains Weight (g)	% of Grains with Holes	Abrasion/Bag *
Initial	216	30.6 ± 0.2 a	30.5 ± 0.9 a	NA
After 7 months				
PICS™ bag	36	30.8 ± 0.2 a	28.1 ± 2.6 a	16.3 ± 8.1 a
AgroZ^®^ bag	36	30.2 ± 0.2 a	29.8 ± 0.5 a	17.0 ± 2.3 a
SuperGrainbag™	36	30.3 ± 0.2 a	29.2 ± 1.0 a	14.0 ± 4.0 a
EVAL™ bag	36	30.2 ± 0.2 a	31.1 ± 1.1 a	11.7 ± 3.2 a
ZeroFly^®^ bag	36	27.3 ± 0.6 b	53.3 ± 4.7 b	NA
Woven bag	36	26.2 ± 0.3 c	62.5 ± 3.0 c	NA
ANOVA		*F* = 26.54; *df* = 6/137; *p* < 0.01	*F* = 44.032; *df* = 6/137; *p* < 0.01	*F* = 0.24; df = 3/11; *p* = 0.865

Means within the same column followed by the same letter are not significantly different (LSD 5%). * *n* for the abrasion (minor holes) is four liners for each brand of hermetic bag.

## References

[1] Mutambuki K., Ngatia C.M., Lorini I., Bacaltchuk B., Beckel H., Deckers D., Sundfeld E., Dos Santos J.P., Biagi J.D., Celaro J.C., Faroni L.R.D., Bortolini L. (2006). Losss assessment of on-farm stored maize in semi arid area of Kitui District, Kenya. Proceedings of the 9th International Working Conference on Stored Product Protection.

[2] Costa S.J. (2014). Reducing Food Losses in Sub-Saharan Africa (Improving Post-Harvest Management and Storage Technologies of Smallholder Farmers).

[3] Abass A.B., Ndunguru G., Mamiro P., Alenkhe B., Mlingi N., Bekunda M. (2014). Post-harvest food losses in a maize-based farming system of semi-arid savannah area of Tanzania. J. Stored Prod. Res..

[4] Gitonga Z.M., De Groote H., Kassie M., Tefera T. (2013). Impact of metal silos on households’ maize storage, storage losses and food security: An application of a propensity score matching. Food Policy.

[5] Tefera T. (2012). Post-harvest losses in African maize in the face of increasing food shortage. Food Secur..

[6] Kumar D., Kalita P. (2017). Reducing Postharvest Losses during Storage of Grain Crops to Strengthen Food Security in Developing Countries. Foods.

[7] Baoua I.B., Amadou L., Ousmane B., Baributsa D., Murdock L.L. (2014). PICS bags for post-harvest storage of maize grain in West Africa. J. Stored Prod. Res..

[8] Njoroge A.W., Affognon H.D., Mutungi C.M., Manono J., Lamuka P.O., Murdock L.L. (2014). Triple bag hermetic storage delivers a lethal punch to *Prostephanus truncatus* (Horn) (Coleoptera: Bostrichidae) in stored maize. J. Stored Prod. Res..

[9] Tubbs T., Baributsa D., Woloshuk C. (2016). Impact of opening hermetic storage bags on grain quality, fungal growth and aflatoxin accumulation. J. Stored Prod. Res..

[10] Williams S.B., Baributsa D., Woloshuk C. (2014). Assessing Purdue Improved Crop Storage (PICS) bags to mitigate fungal growth and aflatoxin contamination. J. Stored Prod. Res..

[11] Kadjo D., Ricker-Gilbert J., Abdoulaye T., Shively G., Baco M.N. (2018). Storage losses, liquidity constraints, and maize storage decisions in Benin. Agric. Econ..

[12] Baributsa D., Abdoulaye T., Lowenberg-DeBoer J., Dabiré C., Moussa B., Coulibaly O., Baoua I. (2014). Market building for post-harvest technology through large-scale extension efforts. J. Stored Prod. Res..

[13] Baributsa D., Njoroge A.W. (2020). The use and profitability of hermetic technologies for grain storage among smallholder farmers in eastern Kenya. J. Stored Prod. Res..

[14] Kitch L.W., Ntoukam G. (1991). Airtight Storage of Cowpea in Triple Plastic Bags (Triple-Bagging).

[15] Murdock L.L., Baributsa D. Hermetic storage for those who need it most-subsistence farmers. Proceedings of the 11th International Working Conference on Stored Product Protection.

[16] Navarro S., Varnava A., Donahaye E., Navarro S., Donahaye E. (1993). Preservation of grain in hermetically sealed plastic liners with particular reference to storage of barley in Cyprus. Proceedings of the International Conference of Controlled Atmosphere and Fumigation in Grain Storages.

[17] Baributsa D., Lowenberg-DeBoer J., Murdock L., Moussa B. Profitable chemical-free cowpea storage technology for smallholder farmers in Africa: Opportunities and challenges. Proceedings of the 10th International Working Conference on Stored Product Protection.

[18] Guenha R., Das Virtudes Salvador B., Rickman J., Goulao L.F., Muocha I.M., Carvalho M.O. (2014). Hermetic storage with plastic sealing to reduce insect infestation and secure paddy seed quality: A powerful strategy for rice farmers in Mozambique. J. Stored Prod. Res..

[19] Mutungi C.M., Affognon H., Njoroge A.W., Baributsa D., Murdock L.L. (2014). Storage of mung bean (*Vigna radiata* [L.] Wilczek) and pigeonpea grains (*Cajanus cajan* [L.] Millsp) in hermetic triple-layer bags stops losses caused by *Callosobruchus maculatus* (F.) (Coleoptera: Bruchidae). J. Stored Prod. Res..

[20] Mutungi C., Affognon H.D., Njoroge A.W., Manono J., Baributsa D., Murdock L.L. (2015). Triple-layer plastic bags protect dry common beans (*Phaseolus vulgaris*) against damage by *Acanthoscelides obtectus* (Coleoptera: Chrysomelidae) during storage. J. Econ. Entomol..

[21] George M.L.C. (2011). Effective Grain Storage for Better Livelihoods of African Farmers Project.

[22] Coffi H., Nyabicha J., Ouma J.O. (2016). The Use of Hermetic Bags for on Farm Storage of Grains and Pulses Against Insect Pests. Outlooks Pest Manag..

[23] AgResults (2018). Kenya on Farm Storage Pilot Project: Airtight (Hermetic) Devices.

[24] De Groote H., Kimenju S.C., Likhayo P., Kanampiu F., Tefera T., Hellin J. (2013). Effectiveness of hermetic systems in controlling maize storage pests in Kenya. J. Stored Prod. Res..

[25] Vales M.I., Ranga Rao G.V., Sudini H., Patil S.B., Murdock L.L. (2014). Effective and economic storage of pigeonpea seed in triple layer plastic bags. J. Stored Prod. Res..

[26] Afzal I., Bakhtavar M.A., Ishfaq M., Sagheer M., Baributsa D. (2017). Maintaining dryness during storage contributes to higher maize seed quality. J. Stored Prod. Res..

[27] Mutambuki K., Affognon H., Likhayo P., Baributsa D. (2019). Evaluation of purdue improved crop storage triple layer hermetic storage bag against *Prostephanus truncatus*/(Horn) (coleoptera: Bostrichidae) and *Sitophilus zeamais* (motsch.) (coleoptera: Curculionidae). Insects.

[28] Singano C.D., Mvumi B.M., Stathers T.E. (2019). Effectiveness of grain storage facilities and protectants in controlling stored-maize insect pests in a climate-risk prone area of Shire Valley, Southern Malawi. J. Stored Prod. Res..

[29] Mlambo S., Mvumi B.M., Stathers T., Mubayiwa M., Nyabako T. (2017). Field efficacy of hermetic and other maize grain storage options under smallholder farmer management. Crop Prot..

[30] Baoua I.B., Amadou L., Lowenberg-DeBoer J.D., Murdock L.L. (2013). Side by side comparison of GrainPro and PICS bags for postharvest preservation of cowpea grain in Niger. J. Stored Prod. Res..

[31] Chigoverah A.A., Mvumi B.M. (2018). Comparative efficacy of four hermetic bag brands against *Prostephanus truncatus* (Coleoptera: Bostrichidae) in Stored Maize Grain. J. Econ. Entomol..

[32] Baoua I.B., Amadou L., Margam V., Murdock L.L. (2012). Comparative evaluation of six storage methods for postharvest preservation of cowpea grain. J. Stored Prod. Res..

[33] Baributsa D., Baoua I., Abdoulaye T., Murdock L.L. (2015). A Guide on the Use of PICS Bags for Grain Storage.

[34] Tiongson R.L., Semple R.L., Hicks P.A., Lozare J.V., Castermans A. (1992). Storage losses and their estimation. Towards Integrated Commodity and Pest Management in Grain Storage.

[35] FAO (2018). Guidelines on the Measurement of Harvest and Post-Harvest Losses: Recommendations on the Design of a Harvest and Post-Harvest Loss Statistics System for Food Grains (Cereals and Pulses).

[36] Murdock L.L., Margam V., Baoua I., Balfe S., Shade R.E. (2012). Death by desiccation: Effects of hermetic storage on cowpea bruchids. J. Stored Prod. Res..

[37] Williams S.B., Murdock L.L., Baributsa D. (2017). Safe storage of maize in alternative hermetic containers. J. Stored Prod. Res..

[38] Walker S., Jaime R., Kagot V., Probst C. (2018). Comparative effects of hermetic and traditional storage devices on maize grain: Mycotoxin development, insect infestation and grain quality. J. Stored Prod. Res..

[39] Paudyal S., Opit G.P., Osekre E.A., Arthur F.H., Bingham G.V., Payton M.E., Danso J.K., Manu N., Nsiah E.P. (2017). Field evaluation of the long-lasting treated storage bag, deltamethrin incorporated, (ZeroFly^®^ Storage Bag) as a barrier to insect pest infestation. J. Stored Prod. Res..

[40] Abass A.B., Fischler M., Schneider K., Daudi S., Gaspar A., Rüst J., Kabula E., Ndunguru G., Madulu D., Msola D. (2018). On-farm comparison of different postharvest storage technologies in a maize farming system of Tanzania Central Corridor. J. Stored Prod. Res..

[41] Shires S.W. (1979). Influence of temperature and humidity on survival, development period and adult sex ratio in *Prostephanus truncatus* (Horn) (Coleoptera, Bostrichidae). J. Stored Prod. Res..

[42] Kharel K., Mason L.J., Williams S.B., Murdock L.L., Baoua I.B., Baributsa D. (2018). A time-saving method for sealing Purdue Improved Crop Storage (PICS) bags. J. Stored Prod. Res..

[43] Williams S.B., Murdock L.L., Baributsa D. (2017). Sorghum seed storage in Purdue Improved Crop Storage (PICS) bags and improvised containers. J. Stored Prod. Res..

[44] Baributsa D., Baoua I.B., Bakoye O.N., Amadou L., Murdock L.L. (2017). PICS bags safely store unshelled and shelled groundnuts in Niger. J. Stored Prod. Res..

[45] Baoua I.B., Bakoye O., Amadou L., Murdock L.L., Baributsa D. (2018). Performance of PICS bags under extreme conditions in the sahel zone of Niger. J. Stored Prod. Res..

[46] Maboudou G.A., Adégbola P.Y., Coulibaly O., Hell K., Amouzou E. Factors affecting the use of improved clay store for maize storage in the central and northern Benin. Proceedings of the 4th International Crop Science Congress: New Directions for a Diverse Planet.

[47] Mutungi C.M., Affognon H.D. (2013). ICIPE Policy Brief No 2/13.

[48] Kitch L.W., Ntoukam G., Shade R.E., Wolfson J.L., Murdock L.L. (1992). A solar heater for disinfesting stored cowpeas on subsistence farms. J. Stored Prod. Res..

[49] Vestergaard ZeroFly® Hermetic. https://www.vestergaard.com/zerofly/zerofly-hermetic/.

[50] Nganga J., Mutungi C., Imathiu S., Affognon H. (2016). Effect of triple-layer hermetic bagging on mould infection and aflatoxin contamination of maize during multi-month on-farm storage in Kenya. J. Stored Prod. Res..

[51] Baributsa D., Djibo K., Lowenberg-DeBoer J., Moussa B., Baoua I. (2014). The fate of triple-layer plastic bags used for cowpea storage. J. Stored Prod. Res..

[52] Baributsa D., Ignacio M.C., Maier D.E. (2020). Developments in the use of hermetic bags for grain storage. Advances in Postharvest Management of Cereals and Grains.

[53] Foy C., Wafula M. (2016). Scaling Up of Hermetic Bag Technology (PICS) in Kenya: Review of Successful Scaling of Agricultural Technologies.

